# Cellular Metabolomics Reveals Differences in the Scope of Liver Protection Between Ammonium-Based Glycyrrhizinate and Magnesium Isoglycyrrhizinate

**DOI:** 10.3390/metabo15040263

**Published:** 2025-04-10

**Authors:** Yihua Zhang, Han Hao, Hui Li, Qiong Duan, Xiaoming Zheng, Yan Feng, Kun Yang, Shigang Shen

**Affiliations:** 1 Key Laboratory of Analytical Science and Technology of Hebei Province, College of Chemistry and Materials Science, Hebei University, Baoding 071002, China; zhangyihua@hbyxjy.org.cn; 2 NDMA Key Laboratory for Quality Control and Evaluation of Generic Drug, Hebei Institute for Drug and Medical Device Control, Shijiazhuang 050200, China; lihui@hbyxjy.org.cn (H.L.); duanqiong@hbyxjy.org.cn (Q.D.); zhengxiaoming@hbyxjy.org.cn (X.Z.); fengyan@hbyxjy.org.cn (Y.F.); 3 School of Pharmacy, Hebei Medical University, Shijiazhuang 050017, China; 24034100605@stu.hebmu.edu.cn

**Keywords:** hepatoprotective, diammonium glycyrrhizinate, magnesium isoglycyrrhizinate, cell metabolomics

## Abstract

**Background:** Despite the well-established liver-protective efficacy of monoammonium glycyrrhizinate (MONO), diammonium glycyrrhizinate (DIAM), and magnesium isoglycyrrhizinate (MAGN), which has been translated into clinical practice, their clinical differentiation remains elusive owing to their structural similarities and overlapping therapeutic effects. **Methods:** The present study delves into the pharmacokinetics, cellular-level liver-protective potencies, and underlying mechanisms of action of these three compounds through a comprehensive analysis. **Results:** The findings reveal that both DIAM and MAGN exhibit superior bioavailability and hepatoprotective profiles compared to MONO. Notably, an investigation of the metabolic pathways mediating liver protection in normal human liver cells (LO2), utilizing an ultra-performance liquid chromatography–time of flight tandem mass spectrometry (UPLC-TOF-MS/MSe) platform, demonstrated that MAGN augments antioxidant components, thereby favoring its application in drug-induced liver injury (DILI). Conversely, DIAM appears to be a more suitable candidate for addressing non-alcoholic fatty liver disease (NAFLD) and viral hepatitis. **Conclusion:** This study contributes novel perspectives on the mechanisms of action and potential clinical utilities of DIAM and MAGN in liver disease prevention and management.

## 1. Introduction

Acute liver injury denotes a clinical syndrome characterized by an abrupt onset of hepatic cellular damage in individuals devoid of pre-existing chronic liver disease. This damage arises from diverse etiologies, encompassing abnormalities in liver function, the development of hepatic encephalopathy, and disruptions in coagulation. It serves as the ubiquitous pathological cornerstone underpinning various acute hepatic disorders encountered in clinical settings. In severe manifestations, acute liver injury can precipitate life-threatening complications, such as liver failure, profound coagulation dysfunction, and the onset of hepatic encephalopathy, necessitating prompt medical intervention [1,2]. Acute liver injury can be primarily attributed to two principal etiologies: viral hepatitis and DILI. Notably, due to the accessibility and prevalent utilization of acetaminophen (APAP) as an over-the-counter medication, often incorporated into various combination formulations, APAP-induced liver injury constitutes the most prevalent form of DILI in the United States and globally. This underscores the significance of vigilant monitoring and awareness surrounding the safe administration and dosage of APAP to mitigate the risk of hepatic toxicity [3,4].

**Figure 1:**
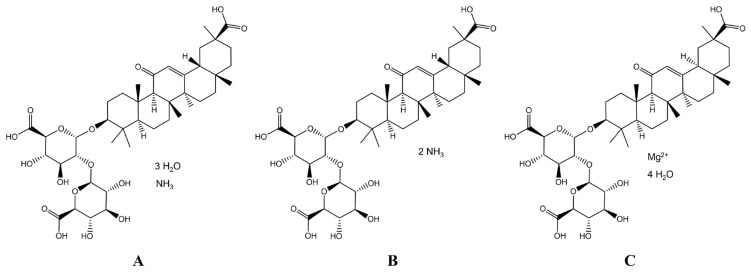
Structural formula of (**A**) MONO, (**B**) DIAM, and (**C**) MAGN.

Numerous natural compounds exhibit a salutary effect on the liver’s health. Licorice (*Glycyrrhiza* spp.) exhibits anti-inflammatory, antiviral, hepatoprotective, antioxidant, and anti-ulcer activities, primarily mediated via NF-κB inhibition, immune modulation, and oxidative stress reduction [5]. Among its myriad of clinical therapeutic interventions, the employment of glycyrrhizinate or isoglycyrrhizinate (Figure 1) for hepatic protection has emerged as a particularly advantageous approach, exhibiting distinctive benefits. Glycyrrhizinate refers to salts or derivatives of glycyrrhizic acid (a triterpenoid saponin), primarily extracted from the roots of licorice plants (*Glycyrrhiza glabra*, *G. uralensis*, or *G. inflata*). It is classified as a natural surfactant and bioactive compound, commonly used in pharmaceuticals (anti-inflammatory and hepatoprotective agents) and cosmetics (with foaming or emulsifying properties) [6], while magnesium glycyrrhizinate can be obtained by the isomerization of glycyrrhizinate under acidic or alkaline conditions [7]. Here, MONO was an ammonium salt preparation of 18α-glycyrrhizinate and DIAM was recognized as a mixture of 18α and 18β, while MAGN, also known as tetrahydrate magnesium, was the 18-α glycyrrhizic acid stereoisomer [8]. MONO, DIAM, and MAGN primarily mitigate intrahepatic inflammatory responses by activating the Nrf-2 pathway, which fortifies liver cell membranes and enhances hepatic function. This cascade of events ultimately leads to reduced liver cell damage, effectively safeguarding the liver and diminishing the deleterious effects of inflammatory reactions on its integrity [8]. Despite their structural similarities, leading to interchangeable clinical use in drug-induced liver injury (DILI), their distinct mechanisms highlight divergent therapeutic pathways, such as MONO’s modulation of Mrp2/Ntcp/Oatp1A4 transporters to counter rifampicin/isoniazid toxicity [9] and IL-10-mediated ROS/TNF-α suppression against LPS damage [10], DIAM’s normalization of ALT/AST levels in CCl_4_-induced metabolic dysfunction [11] and immune regulation via NKT/Treg balance [12], and MAGN’s inhibition of lipid peroxidation in doxorubicin toxicity [13] and p38/JNK pathway-mediated immune injury alleviation [14]. Current methodologies struggle to differentiate their efficacy due to overlapping structural and functional features, as conventional analyses often mask mechanistic distinctions. Clarifying their unique mechanisms is critical for optimizing clinical applications, refining therapeutic thresholds, and advancing targeted interventions in liver injury management.

Conventionally, at the cellular level, a ubiquitous biochemical analysis approach is employed to interrogate the expression profiles of pivotal enzymes via Western blotting and the transcriptional activity of crucial genes utilizing Polymerase Chain Reaction (PCR). This strategy aims to unravel pharmacological mechanisms grounded in cytotoxicity. However, the inherent limitation of biochemical analysis lies in its ability to solely interrogate the compound under investigation, rendering it challenging to detect variations that transcend the predefined scope of examination. Furthermore, the quantitative measurements of enzyme activity obtained through Western blotting do not invariably mirror their biological functionality. Variations in enzyme conformation, amino acid residues, or alterations in their microenvironment can significantly modulate their activity. For instance, the subcellular localization and phosphorylation status of enzymes within cells can profoundly impact their functionality, yet these nuanced differences may escape detection when relying solely on traditional biochemical methods, resulting in seemingly uniform response values [15]. Moreover, subtle alterations in enzyme content, which are often overlooked, can potentially trigger profound shifts in the concentration of small molecule metabolites. These minute variations in enzymatic abundance, though seemingly inconsequential, can significantly modulate metabolic pathways and ultimately impact the overall metabolic landscape [16].

Given the limitations of conventional biochemical assays in discerning subtle mechanistic differences among structurally analogous compounds, advanced omics approaches such as metabolomics are imperative to uncover their distinct therapeutic pathways. Metabolomics, as an emerging branch of systems biology, aims to systematically analyze the dynamic changes in endogenous metabolites in cells, tissues, and body fluids, reveal their regulatory mechanisms under physiological or environmental stimuli, and provide a high-sensitivity perspective for exploring biological metabolic networks [16,17,18]. This technology avoids prior hypothesis interference through unbiased whole metabolite detection and is particularly adept at capturing significant metabolic differences caused by small disturbances in complex biological systems, thereby reflecting the body’s health status and stress response [16]. By combining modeling methods such as Orthogonal Partial Least Squares Discriminant Analysis (OPLS-DA), metabolomics can provide in-depth analysis of the metabolic reprogramming process after drug intervention. Among them, cellular metabolomics provides unique advantages in revealing the potential mechanism of action of weak-acting drugs by accurately identifying differential metabolites related to drug action [19,20,21].

In the present study, a DILI model was meticulously constructed through the administration of excessive APAP to LO2 (normal human hepatocyte) cells. Subsequently, the protective effects of varying types and concentrations of MONO, DIAM, and MAGN against this insult were investigated. Furthermore, the intricacies of the liver-protective mechanisms exhibited by MONO, DIAM, and MAGN were comprehensively evaluated and illuminated through an untargeted metabolomics approach, rooted in the robust UHPLC-Q-TOF-MS/MSe platform complemented by the IDA mode, providing invaluable insights into their therapeutic potential and modes of action.

## 2. Materials and Methods

### 2.1. Chemicals

Methanol (HPLC) was bought from Fisher Chemical (Waltham, MA, USA). Acetonitrile (HPLC-MS grade) was bought from Honeywell (Geismar, LA, USA). Dimethyl sulfoxide (DMSO) was obtained from Tokyo Chemical Industry Co., Ltd. (Tokyo, Japan). 3-(4,5-dimethylthiazol-2-yl)-2,5-diphenyl tetrazolium bromide (MTT) was bought from Beijing Innochem Co., Ltd. (Beijing, China). RPMI 1640 culture medium, fetal bovine serum (FBS), Trypsin Solution A (0.25%), Phosphate-Buffered Saline (PBS) and penicillin–streptomycin (PS) were purchased from Biological Industries (Israel). Double-distilled water was used for all experiments.

### 2.2. Pharmacokinetics

#### 2.2.1. Animal Experiments

Nine male Sprague Dawley rats, each weighing approximately 250 g ± 25 g, were meticulously selected as the experimental subjects (sourced from Beijing SPF Biotechnology Co., Ltd., (Beijing, China) SPF grade, licensed under SCXK (Jing) 2019-0010). The experimental protocol was approved by the Institutional Animal Care and Use Committee of Hebei Medical University and conformed to the Guide for Care and Use of Laboratory Animals (approval NO. 2023040). Prior to and throughout the experimental period, the rats were provided with unrestricted access to standard rodent chow and water. The housing facilities for these animals adhered strictly to the standards outlined by the International Association for the Evaluation and Certification of Laboratory Animal Care, ensuring optimal conditions for the animals’ wellbeing. The experimental feeding environment was maintained at a temperature of 22–24 °C, with a relative humidity of 50 ± 5%, and a 12-h light/dark cycle to mimic natural diurnal rhythms. The rats were randomly allocated into three distinct groups, with an equal distribution of three rats per group. MONO, DIAM, and MAGN were administered orally at a dose of 20 mg/kg. The amount administered was calculated as the contents of glycyrrhizinate or magnesium isoglycyrrhizinate. Each compound was dissolved in 0.5% carboxymethylcellulose sodium (CMC-Na) solution and delivered immediately to rats via gastric gavage. Blood samples of 0.2 mL were obtained at various time points post-administration (0.5, 1, 2, 4, 8, 12, 24, and 48 h) and centrifuged at 1500× *g* for 10 min at 4 °C, allowing for the separation of plasma, which was collected and stored at −20 °C for subsequent analysis.

#### 2.2.2. HPLC Method

Fifty microliters (50 μL) of plasma was obtained and spiked with 10 μL of dipyridamole solution as an internal standard. Subsequently, 100 μL of acetonitrile was added to precipitate the proteins, followed by vortex mixing. The mixture was centrifuged at 6000× *g* for 10 min at 4 °C. The resulting precipitate was discarded, and the supernatant was evaporated to dryness under a nitrogen stream at 60 °C. The residual precipitate was redissolved in 200 μL of mobile phase, vortexed for 5 min, and sonicated for 30 s. Following this, the solution was centrifuged again at 6000× *g* for 10 min at 4 °C. Then, 10 μL of the supernatant was injected and the chromatogram was recorded for analysis.

HPLC was employed using the Agilent 1200 series system (Agilent Technologies, Wilmington, DE, USA) with Agilent Chemstation LC B.04.03 Software. The separation was on a Venusil MP C18(2) chromatographic column (150 × 4.6 mm, 5 μm, Agela Technologies, Tianjin, China) based on the United States Pharmacopeia with adjustments performed. Isocratic elution with a mixture of 40 volumes of acetonitrile and 60 volumes of water (containing 0.5% acetic acid) was used. The flow rate and temperature were 1.0 mL/min and 40 °C, respectively. A volume of 10 μL of the sample was injected under 254 nm for analyses and with three repetitions for each test (*n* = 3). All the pharmacokinetic data were calculated by Drug and Statistics (DAS) 2.0 software (Mathematical Pharmacology Professional Committee of China, Shanghai, China) and presented as mean ± SD. The main parameters such as the area under the curve (AUC), the maximum plasma concentration (*C*_max_), the time to *C*_max_ (*T*_max_), and elimination half-life (*t*_1/2_) were tabulated.

### 2.3. Cell Viability

Cell culture and model construction: The experimental LO2 cells were provided by Shanghai Fuheng Biotechnology Co., Ltd. (Shanghai, China). Cells induced by APAP formed a cell model of liver injury. The cells were cultured in RPMI 1640 medium containing 100 units of penicillin, 100 μg/mL streptomycin, and 10% FBS at 37 °C and 5% CO_2_ atmosphere. The cells were changed daily and passaged when the contact reached 80%. Cells in the logarithmic growth phase were seeded onto a 96-well plate at a density of 4000 cells per well and subsequently incubated at 37 °C in an atmosphere containing 5% CO_2_. After 24 h of pre-incubation, cells were exposed to APAP at concentrations of 2, 4, 8, and 16 mM, followed by continued culture for the designated time periods. Subsequently, 20 μL of MTT solution (5 mg/mL, dissolved in PBS) was added to each well and incubated for an additional 4 h at 37 °C. To dissolve the formed formazan crystals, 200 μL of DMSO was added. The absorbance values were then measured at 490 nm using a microplate reader (INFINITE 200 PRO, Tecan, Männedorf, Switzerland). The cell survival rates at different APAP concentrations were calculated, and a cell survival rate below 60% was considered indicative of successful model construction. The specific formula employed for calculating the cell survival rate is provided below. Based on the results, the final concentration of APAP inducing the desired effect was determined to be 16 mM.

$$Cell\;viability=\frac{A_{APAP}-A_{Blank}}{A_{Control}-A_{Blank}}$$

Assessment of hepatocyte-protective effects: Cells in the logarithmic growth phase were seeded onto a 96-well plate at a density of 4000 cells per well, followed by incubation at 37 °C in an atmosphere containing 5% CO_2_. After 24 h of incubation, cells were treated with 16 mM APAP, followed by the addition of MONO, DIAM, and MAGN at three escalating concentrations (200, 400, and 600 μM for each compound). The cell survival rate was quantitatively evaluated using MTT method. The protective efficacy of the compounds was then calculated employing the formula outlined below.

(1) $$Hepatocytes\;protection\;rate=\frac{A_{Glycyrrhizinate}-A_{APAP}}{A_{normal}-A_{APAP}}\times 1$$

### 2.4. Cell Metabolomics

#### 2.4.1. Measurement Conditions

LC-MS/MS analysis was conducted utilizing a Waters Acquity H UPLC system, interfaced with a Q-MS detector (Xevo G3 QTof, Waters, Milford, MA, USA). Chromatographic separation of metabolites was achieved on a BEH C18 Column (1.7 µm, 2.1 mm × 100 mm, Waters), maintained at a temperature of 40 °C. The injector sample chamber was maintained at 4 °C to ensure optimal sample handling. For positive ion analysis, the mobile phase comprised two solvents, 0.1% formic acid in water (solvent A) and acetonitrile (solvent B), delivered at a flow rate of 300 μL/min. The elution gradient was programmed as follows (acetonitrile percentage): 0–1 min 5%, 1–2 min 5–30%, 2–4 min 30–50%, 4–16 min 50–90%, 19–21 min 95%, 21–21.1 min 95–5%, and 21.1–23 min 5%. The capillary, sample cone, and source offset were set as 2.18 kV, 40, and 30. The temperatures of the source and desolvation were 100 and 600 °C. The gas flow of the cone gas and desolvation gas was set as 50 and 800 L/h. For the negative ion mode, the mobile phase consisted of 10 mM ammonium acetate (solvent A) and acetonitrile (solvent B), delivered at a flow rate of 300 μL/min. The elution gradient was programmed as follows: 0–1 min, 5%; 1–3 min, 5–80%; 3–4 min, 80–82%; 4–6 min, 82–88%; 6–25 min, 88–92%; 25–27 min, 92–95%; 27–27.1 min, 95–5%; 27.1–29 min, 5%. The mass spectrometry conditions were similar to those employed in the positive mode, with the exception of the capillary voltage, which was set to 2 kV. All samples were analyzed using an ESI source in MSe full scan mode, with a mass range spanning from *m*/*z* 50 to 2000 and a scan time of 0.5 s. Additionally, an automatic calibration system was employed to automatically adjust the MS and MSe parameters every 10 s.

#### 2.4.2. Sample Preparation

LO2 cells in the logarithmic growth phase were seeded into a 6-well plate at a density of 3 × 10^4^ cells per well and incubated overnight under conditions of 37 °C and 5% CO_2_. Subsequently, varying concentrations of APAP were introduced, and MONO, DIAM, and MAGN were dissolved in the culture medium prior to their addition to the wells, ensuring a final concentration of 600 μM. The cells were then exposed to these samples for 48 h, following which the culture medium was discarded. The cells were rinsed with pre-cooled PBS at 4 °C and subsequently inactivated by the addition of 80% methanol maintained at a temperature below −20 °C. The cells were scraped off and homogenized using a cell disruptor for 3 min in an ice bath. The homogenized samples were centrifuged at 7500× *g* for 20 min at 4 °C, and the resultant supernatant was collected and dried using nitrogen gas. The dried samples were redissolved in 80% methanol at −20 °C, vortexed for 1 min, sonicated for 30 s, and then centrifuged again at 7500× *g* for 20 min at 4 °C. A total of 10 μL was allocated from each sample and mixed as a quality control (QC) sample, while 5 μL of the supernatant was subjected to UPLC-MS/MSe data acquisition on the instrument. The entire procedure was repeated in parallel for all samples, with a total of six replicates performed.

#### 2.4.3. Data Reliability Analysis

To ensure the stability and reproducibility of the instrumentation, QC samples were systematically integrated within the sequence of test analytes and administered at regular intervals of every six analytes. Furthermore, an investigation into the stability and the duration of this stability for the samples was conducted, aiming to validate the acquisition of reliable data. Employing an unsupervised, multivariate statistical approach, specifically the principal component analysis (PCA) algorithm, based on comprehensive sample set from both the control and treatment groups, enabled the identification of significant differences, if any, between these groups. Additionally, the extent of clustering among QC sampling points served as an indicator of the instrument’s stability, providing a quantitative measure of its performance consistency.

#### 2.4.4. Metabolomics Data Analysis

Firstly, the raw data in the “.raw” format were imported into Progenesis QI software (Waters, Milford, MA, USA). This processing encompassed peak alignment, deconvolution, denoising, and other essential steps, resulting in the extraction of crucial information including retention time, charge-to-mass ratio, compound names, peak areas, and related parameters. To ensure data comparability and accuracy, each peak was then normalized against the total ion intensity, yielding a standardized matrix of retention time, peak area, and *m*/*z* values. Compounds exhibiting a variation exceeding 30% in QC samples were excluded from further analysis. Concurrently, the processed data were imported into SIMCA-P 14.0 software (Umetrics, Umeaa, Sweden) for advanced multivariate statistical analysis, encompassing principal component analysis (PCA) and supervised Orthogonal Partial Least Squares Discriminant Analysis (OPLS-DA). Subsequently, the raw data were analyzed by Progenesis QI software for independent-samples t-tests and fold change (FC) tests, yielding *p*-values and FCs to assess the statistical the significance of differences between variables. These methods aim to maximize inter-group differences while deriving Variable Importance in Projection (VIP) values, which quantify the relative contribution of individual variables to the observed differences. PCA was primarily utilized to derive information that adequately represents the original variables through mathematical dimensions, facilitating data compression and dimensionality reduction. In contrast, OPLS-DA, grounded in supervised discriminant analysis, screened for statistically significant differences in variables between groups and assigned VIP values. Metabolites fulfilling the criteria of *p* < 0.05, FC ≥ 1.2 or FC ≤ 0.8, and VIP > 1 were selected as differential biomarkers. Additionally, the quality of the OPLS-DA model was evaluated by assessing the coefficients (R^2^X, R^2^Y, and Q^2^), which estimate the goodness of fitness and predictive capability. Specifically, R^2^X and R^2^Y reflect the interpretability of the X and Y matrices, respectively, within the constructed model. Typically, R^2^ gauges the model’s fitting ability, while Q^2^ assesses its predictive power. The performance of the model was further validated through 20 permutation tests, confirming the absence of overfitting.

Utilizing peak area as the primary analytical unit and applying the criteria of *p* < 0.05, FC > 1.2, and VIP > 1, we screened for signals indicative of differential metabolites. To ensure precision, the relative molecular weight error was set to less than 5 ppm. Subsequently, the accurate mass-to-charge ratios and secondary mass spectrometry fragments were analyzed using Chemspider, in conjunction with databases such as HMDB (http://www.hmdb.ca/, accessed on 15 April 2024), KEGG (https://www.kegg.jp/, accessed on 15 April 2024), and METLIN (http://metlin.scripps.edu/, accessed on 15 April 2024). This approach facilitated the structural deduction of compounds, where the theoretical values in the databases were compared against the MS/MSe experimental data obtained from Progenesis QI to verify the structural identities and ascertain the chemical names of the identified metabolites. Furthermore, leveraging MetaboAnalyst 6.0, a cluster heatmap was generated to visually represent the distribution patterns of the identified differential metabolites, providing a comprehensive overview of their relative abundances and variations.

#### 2.4.5. Pathway and Enrichment Analysis

To elucidate the cytotoxicity mechanisms associated with MONO, DIAM, and MAGN, an analysis of potential biomarkers was performed utilizing the online software platform MetaboAnalyst 6.0 in conjunction with the comprehensive KEGG and RaMP-DB online database. This approach aimed to decipher the underlying biological processes and interactions that may be modulated by these compounds, thus offering insights into their toxicity mechanisms.

### 2.5. Protein Expression

The expression levels of glutamylcysteine synthetase (GCL), glutathione synthetase (GS), and 11β-hydroxysteroid dehydrogenase type 1 (11β-HSD1) in cells were quantified using commercially available enzyme-linked immunosorbent assay (ELISA) kits (Shanghai Aimeng Youning Biotechnology Co., Ltd., Shanghai, China). Cells in the logarithmic growth phase were seeded into 6-well plates at a density of 3 × 10^4^ cells per well, pre-incubated for 24 h, and subsequently exposed to 16 mM APAP with concurrent administration of MONO, DIAM, and MAGN at a final concentration of 600 μM for 48 h. Following three PBS washes, cells were harvested using cell scrapers. Total protein content was determined by the Bradford assay, and samples were normalized to equivalent protein concentrations prior to quantitative analysis of target proteins according to the manufacturer’s protocols.

### 2.6. Statistical Analysis

To rigorously assess the differences in cell survival rate, liver cell protection rate, and pharmacokinetic parameters, a one-way ANOVA was performed using SPSS 26 software (IBM, Chicago, IL, USA). The VIP values of differential metabolites were analyzed using SIMCA-P version 14.0 (Umetrics, Umeaa, Sweden), and metabolites were analyzed using t-tests and FC tests using Progenesis QI. A probability level of 0.05 was considered the minimum significance level.

## 3. Results and Discussion

### 3.1. Pharmacokinetics Analysis

**Figure 2:**
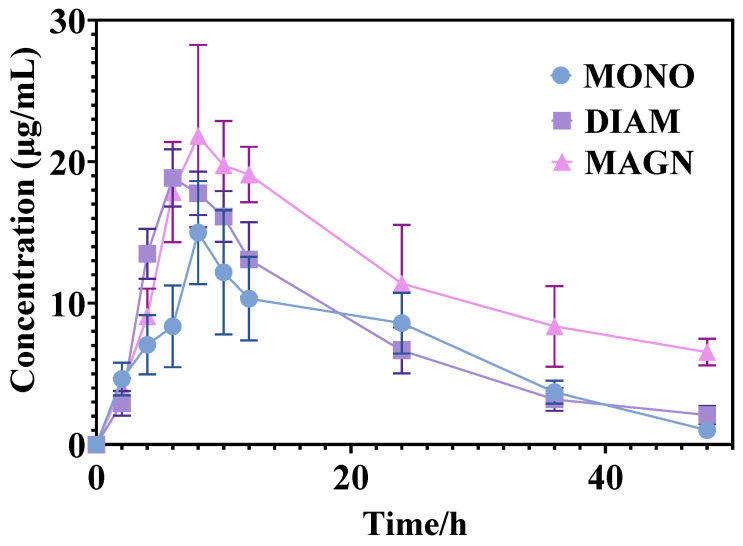
Plasma concentration–time curve of MONO, DIAM and MAGN. Data are presented as means ± standard deviations.

Given the scarcity of suitable in vitro research methodologies, accurately predicting the bioavailability disparities among drugs with comparable physicochemical properties remains a challenge. Nevertheless, the therapeutic efficacy of drugs is intimately tied to their pharmacokinetic profiles. Therefore, the bioavailability of MONO, DIAM, and MAGN was evaluated through oral gavage administration. The concentration profiles for MONO, DIAM, and MAGN in plasma are depicted in Figure 2, while the comprehensive pharmacokinetic parameters derived using DAS 2.0 software are tabulated in Table 1. Upon analysis, it is evident that following a single dose of each drug, MONO, DIAM, and MAGN exhibit comparable pharmacokinetic traits. However, MONO displays the lowest plasma concentration, with an AUC_0-t_ of 320.23 ± 78.63 μg·h·mL^−1^, whereas MAGN attains the highest plasma concentration, with an AUC_0-t_ of 552.31 ± 119.93 μg·h·mL^−1^ (*p* < 0.05). Notably, DIAM, owing to its superior solubility, attains its peak blood concentration (*C*_max_) in just 6 h, outpacing MONO’s 8 h and MAGN’s 9.66 h, aligning with our initial predictions. Intriguingly, despite its oral administration to rats, MAGN exhibits a larger AUC in plasma, which may stem from its reduced solubility, facilitating a slower in vivo release. This observation is further corroborated by its longer half-life (*t*_1/2_) parameter as reported in Table 1. These findings underscore the complexity of predicting and interpreting drug bioavailability, necessitating a multifaceted approach that incorporates in vivo evaluations.

Indeed, the phenomenon where the AUC of insoluble salts surpasses that of soluble salts is not unprecedented. The utilization of insoluble salts constitutes a well-established strategy for achieving slow and controlled drug release. By harnessing the insolubility of active pharmaceutical ingredients (APIs), this approach effectively inhibits their rapid release. Moreover, even among drugs sharing identical chemical compositions, variations in their physical states can lead to significant differences in solubility, dissolution rate, and transmembrane permeability. For instance, Xiao et al. [22] demonstrated that the cocrystal formation of propylthiouracil (PTU) with ligands resulted in a decrease in solubility, dissolution rate, and transmembrane permeability compared to PTU in its pristine form or when paired with different ligands. Notably, the solubility of the PTU-KA cocrystal was found to be merely 15% of that of PTU alone, yet paradoxically, it exhibited superior bioavailability, highlighting the intricate interplay between solubility and bioavailability in the context of drug formulations.

### 3.2. Cell Viability Analysis

**Figure 3:**
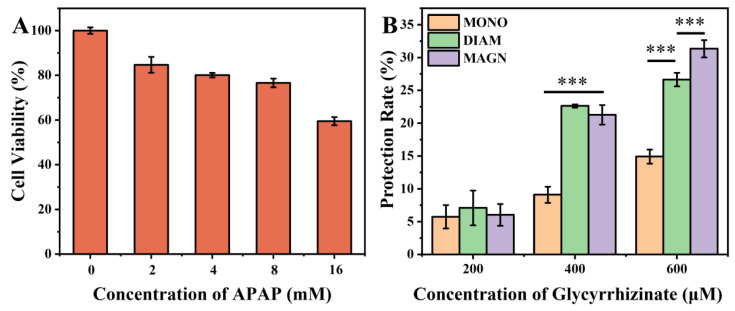
(**A**) Cell viability exposure to APAP for 48 h (*n* = 6). (**B**) Hepatocytic protection of variety glycyrrhizinate. Data are presented as means ± standard deviations (*n* = 6). *** *p* < 0.001. One-way ANOVA with Tukey’s post hoc test.

To assess the therapeutic efficacy of MONO, DIAM, and MAGN in mitigating DILI, a liver injury model was established through the induction of LO2 cells with APAP. Subsequently, the hepatoprotective effects of these compounds were evaluated post-treatment. The findings revealed that, following 24 h of APAP exposure, a concentration-dependent cytotoxicity towards LO2 cells was evident, with cell survival rates dipping below 60% at APAP concentrations exceeding 16 mM, confirming the successful establishment of the liver injury model (Figure 3A).

Upon treatment with varying concentrations of MONO, DIAM, and MAGN, concentration-dependent therapeutic effects were discernible (Figure 3B). Notably, at moderate doses (400 μM), DIAM and MAGN exhibited comparable liver protection rates, which were statistically indistinguishable and surpassed that of MONO. However, as the concentration escalated to 600 μM, MAGN demonstrated a superior protective rate compared to DIAM. This enhanced protective capacity of MAGN over MONO and DIAM may stem from its robust affinity for cellular membrane lipids, conferring membrane stabilization, an attribute lacking in MONO and DIAM [23]. In the treatment of liver injury, protecting the liver cell membrane is the core strategy for maintaining liver functional integrity and promoting repair. When the liver cell membrane is damaged by factors such as viruses, alcohol, drugs, or oxidative stress, an increase in its permeability can lead to two serious consequences [24]: on the one hand, the outflow of intracellular substances can disrupt the homeostasis of the microenvironment and trigger DAMP-mediated inflammatory responses with ROS release [25,26], and on the other hand, the dysfunction of membrane surface receptors and transporters directly affects the function of hepatocytes, and even accelerates the apoptosis or necrosis of hepatocytes [27]. Furthermore, MAGN’s favorable lipid solubility and enhanced membrane permeability endow it with a more potent capacity to inhibit reactive oxygen species (ROS) generation [28]. Additionally, MAGN’s prolonged half-life ensures the maintenance of therapeutic concentrations for an extended duration, thereby contributing to its superior therapeutic outcome compared to DIAM and MONO (Figure 3A).

### 3.3. Data Reliability of Analysis

To elucidate the underlying causes of therapeutic disparity among these drugs, the impacts of these drugs on LO2 cells within a liver injury model were scrutinized using metabolomics approaches, notably UPLC-MS/MSe. Under rigorously optimized conditions, the samples were scanned in both positive and negative ion modes to capture a comprehensive metabolic profile. To bolster data reliability, quality control (QC) samples were systematically injected throughout the analytical process. PCA, an unsupervised multivariate analytical tool, was harnessed for the initial interrogation of MS datasets and visualization of grouping patterns.

**Figure 4:**
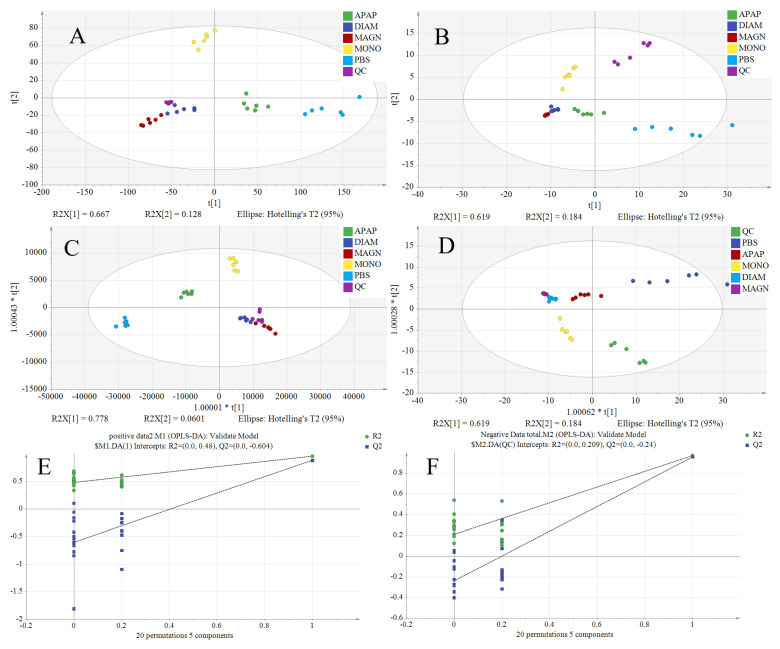
PCA score diagram in positive ion mode (**A**) and negative mode (**B**); OPLS-DA plots in positive ion mode (**C**) and negative mode (**D**); 20 permutation tests in PBS, APAP, MONO, DIAM, and MAGN in positive ion mode (**E**) and negative mode (**F**).

Compared to supervised analysis methods, PCA only reduces dimensionality based on the variance of the data themselves, without relying on sample grouping information, and can more objectively reflect the natural distribution structure of the data [29,30]. Therefore, the magnitude of separation between points in the PCA score plots (Figure 4A,B) reflects the disparity in the genuine metabolic states of cells induced by distinct samples. Here, the x- and y-axes signify the primary and secondary principal components, respectively, derived from dimensionality reduction via PCA. The points in Figure 4A,B represent the projection of the sample on a two-dimensional plane, and the projection position of each point depends on the mass spectrometry data collected for each sample, that is, the metabolite dataset contained in each sample. Samples with similar physiological or pathological states are often composed of metabolites with similar types and contents, so they tend to be located closer to each other on the score graph. The farther the distance between each point, the greater the difference in their physiological and pathological states [30]. Here, the x- and y-axes signify the primary and secondary principal components, respectively, derived from dimensionality reduction via PCA. Notably, the clustering of QC points post-PCA processing underscores the stability of experimental conditions across the entire sampling period. However, despite PCA’s utility, certain groups failed to exhibit a discernible separation trend, prompting the adoption of OPLS-DA for pattern recognition analysis. OPLS-DA, a supervised multivariate statistical approach, adeptly eliminates variations in the independent variable x unrelated to the categorical variable y, thereby enhancing the aggregation of categorical information within the principal component space. Furthermore, OPLS-DA simplifies the model, yielding more pronounced classification and visualization effects compared to PCA. The OPLS-DA score plot (Figure 4C,D) reveals a distinct clustering of MONO, DIAM, and MAGN sample points, indicative of metabolic differences among these groups. Validation through 20 permutation tests affirms the model’s robust fitting and predictive capabilities. Conventionally, R^2^ and Q^2^ values exceeding 0.5 signify a well-fitted and predictive model. Additionally, the intersection of the Q^2^ regression line with the vertical axis below zero validates the model’s efficacy and absence of overfitting. As depicted in Figure 4E,F, all 20 permutation test results adhere to the aforementioned validity criteria, underscoring the congruence between the model’s fitting and predictive abilities. Notably, R^2^ values consistently surpass 0.9, while Q^2^ values exceed 0.8, demonstrating the exceptional performance of the OPLS-DA model in predicting the data matrix (Table 2). The Q^2^ regression line’s intersection with the negative vertical axis underscores the model’s effectiveness and robustness, devoid of overfitting concerns.

### 3.4. Analysis of Differential Metabolites

Given the superior performance of DIAM and MAGN compared to MONO, attention was focused on elucidating the differences between DIAM and MAGN. To achieve this, the OPLS-DA model was employed for analysis. Variables were considered potential biomarkers when they met the criteria of VIP value > 1, *p* < 0.05, and FC > 1.2 or < 0.8. Subsequently, differential compounds were identified by matching MS/MS data and retention time with online databases, including HMDB, METLIN, and KEGG. Ultimately, a total of 46 endogenous metabolites were selected as potential differential biomarkers for distinguishing between the MAGN and DIAM groups (Table 3).

**Figure 5:**
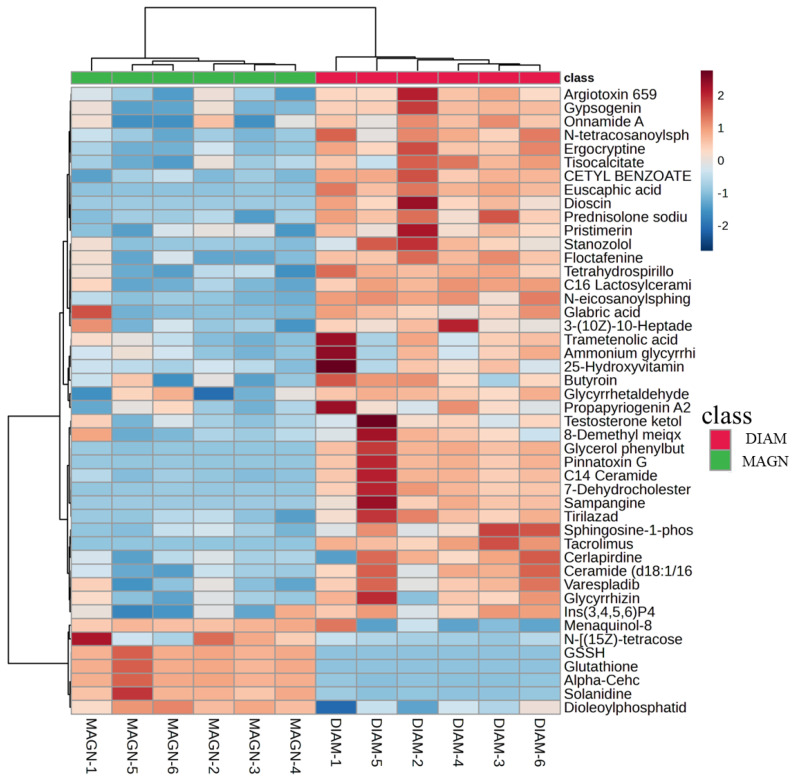
Clustering heatmap of 46 potential biomarkers in three groups. Rows: samples; columns: biomarkers.

Utilizing the hierarchical clustering heatmap function of MetaboAnalyst 6.0 software, the differential metabolites between MAGN and DIAM were visualized (Figure 5). The names of the differential metabolites are indicated on the right, while the sample classifications are marked at the top. In the heatmap, each row represents the relative content of the same metabolite across different samples, and each column represents the quantity of different metabolites within the samples. The results reveal that the levels of these metabolites differed between MAGN and DIAM, with higher metabolite levels being closer to the red color and lower levels being closer to the blue color. The tree diagram on the left depicts the distinct clustering analysis results of different samples. These two related substances exhibited a high correlation, as evidenced by the similar color distribution within each group but differing color distribution between MAGN and DIAM, indicating that MAGN and DIAM induced distinct metabolomic features in LO2 cells when compared to each other.

### 3.5. Metabolic Pathways and Enrichment Analysis

**Figure 6:**
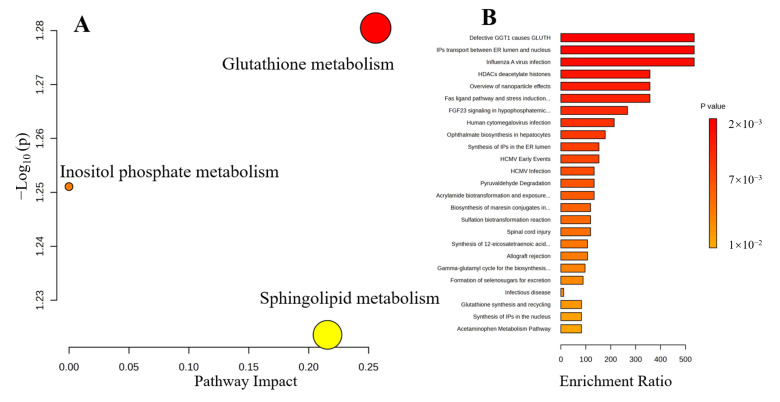
Pathway analysis (**A**) and enrichment analysis (**B**) of DIAM and MAGN.

Given the comparable therapeutic effects observed for ammonium glycyrrhizinate drugs, this study opted not to delve into the distinctions between APAP and MAGN but rather employed the OPLS-DA methodology to explore the disparities between DIAM and MAGN. Figure 6A offers a consolidated overview of the pathway analysis, wherein disparate points signify distinct metabolic pathways. Typically, larger areas and positions nearer to the upper right quadrant signify metabolic pathways of greater significance. In the case of DIAM and MAGN, three pivotal pathways emerged: Glutathione Metabolism, Inositol Phosphate Metabolism, and Sphingolipid Metabolism. Furthermore, Figure 6B presents the outcomes of the enrichment analysis, which highlights the differences between DIAM and MAGN encompassing diverse cellular phenomena, including nucleus endoplasmic reticulum transport, histone deacetylation, and viral infection.

#### 3.5.1. Antioxidant and Anti-Inflammatory Properties

The significance of antioxidants and anti-inflammatory agents in safeguarding liver health cannot be overstated. The liver, a pivotal metabolic organ within the human anatomy, is inherently vulnerable to insults emanating from a myriad of drugs and detrimental lifestyle practices, notably oxidative stress and inflammatory cascades. Consequently, the pursuit of antioxidant and anti-inflammatory strategies has evolved into two paramount avenues for preserving and promoting hepatic wellbeing [31], underscoring their indispensable roles in mitigating the deleterious effects of these insults.

Glutathione, a tripeptide encompassing thiol-containing amino acids, namely glutamic acid, cysteine, and glycine, is ubiquitous within cellular milieus. It assumes a pivotal role not only as a vital antioxidant in the body but also as a participant in a diverse array of cellular metabolic processes [32]. Notably, glutathione exists in both oxidized (GSSG) and reduced (GSH) forms, each with distinct functionalities. GSSG proficiently scavenges free radicals, safeguarding cells from oxidative harm [33], while contributing significantly to cardiovascular health promotion, anti-tumor activities, and the retardation of aging processes. GSH, on the other hand, possesses robust antioxidant capabilities, eliminating oxygen-derived free radicals and safeguarding vital organs such as the liver from oxidative insults [34]. In the context of hepatic protection, GSH exerts inhibitory effects on fatty liver development and alleviates symptoms associated with various liver ailments, including toxic and infectious hepatitis [35]. In the present study, APAP was employed as an inducer of LO2 cell demise, with its mechanism rooted in its hepatic metabolism to yield the intermediary metabolite N-acetyl benzoquinone imine (NAPQI). Although NAPQI inherently possesses a degree of toxicity, it is effectively sequestered by GSH in the liver to form non-toxic complexes, subsequently eliminated via urinary excretion [36]. However, upon excessive APAP administration, NAPQI production escalates, potentially overwhelming the GSH reserves in the liver. This scenario leads to unbound NAPQI molecules directly interacting with hepatic cellular macromolecules, triggering a cascade of detrimental responses, including liver cell necrosis and increased cell membrane permeability, ultimately culminating in liver cell damage and potentially severe consequences like liver failure. Remarkably, LO2 cells subjected to MAGN induction exhibited heightened concentrations of both GSH (1.24 fold than DIAM, *p* < 0.05) and GSSG (1.28 fold than DIAM, *p* < 0.05) in comparison to those treated with DIAM, while maintaining a stable GSH-to-GSSG ratio (Table 3). This finding underscores MAGN’s superior ability to augment GSH reserves, thereby bolstering liver cells’ metabolic competence in neutralizing toxic metabolites without compromising their functional integrity.

Alpha-carboxyethylhydroxychroman (alpha-Cehc) is a water-soluble metabolite of alpha-tocopherol (α-TOH) with potential antioxidant activity [37]. Upon induction with MAGN, LO2 cells manifested heightened levels of alpha-Cehc in comparison to those treated with DIAM, reaching 1.21 times (*p* < 0.001, Table 3). This elevation in alpha-Cehc concentration serves as one of the underlying factors contributing to the reduced mortality rate observed in LO2 cells exposed to MAGN. The enhanced production of alpha-Cehc, a marker of antioxidant status, underscores the ability of MAGN to potentiate the antioxidant defenses within the cells, thereby mitigating the detrimental effects of oxidative stress and ultimately contributing to improved cell survival.

25-Hydroxyvitamin D2, a pivotal bioactive component of vitamin D, has been extensively documented to modulate hepatic inflammation in obese individuals [38]. Its metabolic derivatives, inclusive of 25-hydroxyvitamin D2, are implicated in mitigating inflammatory responses in liver pathologies, notably non-alcoholic fatty liver disease (NAFLD) [39]. Furthermore, vitamin D has demonstrated efficacy in mitigating liver injury in patients afflicted with viral hepatitis [40]. Dioscin, chemically designated as diosgenyl 2,4-di-O-α-L-rhamnopyranosyl-β-D-glucopyranoside, is a significant constituent of several traditional Chinese medicinal formulations, renowned for its diverse biological activities [41]. Notably, dioscin has exhibited promising therapeutic potential in addressing NAFLD [42] and viral hepatitis [43] that often arise as complication of obesity. In the current study, DIAM-induced LO2 cells demonstrated significantly increased concentrations of 25-hydroxyvitamin D2 and dioscin, reaching 3.85-fold and over 10-fold elevations, respectively, compared to MAGN-treated cells, with both comparisons showing statistical significance at *p* < 0.001 (Table 3). These findings indicate that DIAM may possess superior therapeutic potential over MAGN for mitigating hepatic injury associated with viral pathogens or metabolic dysfunction in obesity. This finding underscores the potential of DIAM in harnessing the protective mechanisms mediated by these bioactive molecules, thereby contributing to liver health and function.

#### 3.5.2. Immune Regulation

The liver, as the most extensive reticuloendothelial phagocytic system in humans, plays its pivotal role in immune regulation and phagocytosis, thereby influencing overall immune function. The intricate interplay between liver immune regulation and liver injury underscores the significance of maintaining a delicate balance in immune homeostasis. Imbalances in liver immune regulation can lead to pathological outcomes, including liver injury [44]. Immune imbalance may manifest as either exaggerated or inadequate immune responses, with the former often resulting in excessive infiltration of immune cells and the release of proinflammatory factors. In scenarios such as viral infections, DILI, or autoimmune liver diseases, the immune response may become overly robust, culminating in liver cell damage, inflammation, fibrosis, and ultimately, cirrhosis [45]. Tacrolimus, an effective immunosuppressant, is routinely utilized to modulate autoimmune responses post-organ transplantation [46] and has also demonstrated efficacy in managing autoimmune liver injury [47] and viral-induced liver damage [48]. In parallel, prednisolone sodium tetrahydrothalic, a glucocorticoid, exhibits potent anti-inflammatory and immunosuppressive properties [49]. The present study observed notable variations in the levels of tacrolimus (more than 10-fold) and prednisolone sodium tetrahydrothalic (1.66 fold) in cells induced by DIAM versus MAGN (Table 3), indicating that DIAM may hold greater promise in the context of autoimmune hepatitis or liver injury compared to MAGN. This finding underscores the potential therapeutic advantages of DIAM in modulating immune responses and mitigating liver damage. Notably, although tacrolimus and prednisolone are traditionally considered exogenous drugs, their trace presence in cells may be due to the metabolic derivatives of endogenous analogues, such as tacrolimus possibly producing trace derivatives through the bypass metabolism of cytochrome P450 enzymes [50], while prednisolone may be produced by abnormal branches of endogenous steroid metabolic pathways (such as cholesterol → pregnenolone → cortisol), especially under stress conditions [51]. In addition, the metabolic transformation of DIAM/MAGN or other exogenous precursors included in commercial-grade culture media by cells may also generate these two substances, such as the complex chemical structure of the glycyrrhetinic acid derivative DIAM/MAGN, which may provide reaction substrates. Similar situations have been reported in the research of other scholars [19,52].

#### 3.5.3. Sphingolipids and Ceramides Metabolism

Sphingolipids and ceramides demonstrate a wide range of biological activities. Notably, DIAM significantly enhances the expression of Sphingosine-1-phosphate (d16:1) in LO2 cells, with a 3.78-fold increase compared to MAGN (Table 3), which induces a potent inflammatory reaction through the activation of NOD1/2 receptors, mediated by the NF-κB signaling cascade, ultimately leading to aggravated hepatic damage [53]. In addition, structural analogues such as Sphingosine d18:1 and Sphingosine-1-phosphate (d16:1) contribute to liver damage by inhibiting HIF-2α within macrophages, further emphasizing their deleterious effects [54]. Moreover, C14 Ceramide and C16 Lactosylceramide (d18:1/16:0) were increased by 1.20- and 1.24-fold over MAGN (Table 3). Those have been identified as inhibitors of the PI3K/AKT pathway, which is crucial for sustaining hepatocyte vitality and proliferation, thereby implying their unfavorable roles in maintaining liver health [55,56]. The expression levels of Ceramide d18:1/16:0 and Ins(3,4,5,6)P4 induced by DIAM were higher than those induced by MAGN (Table 3), which exert mechanistically distinct yet synergistic effects in hepatitis progression. Ceramide d18:1/16:0 directly activate mitochondrial apoptotic pathways through Bax oligomerization and cytochrome c release [57], thereby amplifying oxidative stress-induced hepatocyte apoptosis. In addition, Ins(3,4,5,6)P4 aggravates hepatic inflammation by competitively inhibiting IP3 receptor-mediated calcium signaling, which disrupts hepatocyte regeneration and promotes Kupffer cell activation [58]. In summary, with respect to the inositol phosphate and sphingolipid signaling pathways, DIAM does not confer any superiority over MAGN.

In conclusion, while both DIAM and MAGN have demonstrated efficacy in safeguarding hepatic function, there exist discernible variations in their protective modalities and underlying mechanisms. Specifically, MAGN has emerged as a more viable option for mitigating DILI, notably APAP-induced DILI, whereas DIAM has shown promise in addressing NAFLD and viral hepatitis, highlighting their tailored suitability for distinct pathological conditions.

### 3.6. Protein Expression Analysis

**Figure 7:**
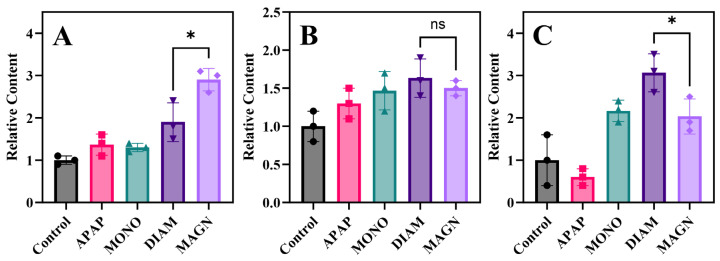
Relative content of GCL (**A**), GS (**B**), and 11 β-HSD1 (**C**) in cells. Data are presented as means ± standard deviations (*n* = 3), * *p* < 0.05, one-way ANOVA with Dunnett’s post hoc test.

To validate the findings in metabolomics, ELISA was used to detect GCL, GS, and 11 β-HSD1 in cells. Specifically, GCL mediates the conjugation of glutamate and cysteine to form γ-glutamylcysteine (γ-GC), representing the rate-limiting step in GSH production [59]. Subsequently, GS catalyzes the glycine incorporation into γ-GC to complete GSH formation [59]. As illustrated in Figure 7A,B, MAGN induction significantly elevated intracellular GCL levels (*p* < 0.05), whereas GS expression remained comparable to DIAM-treated control. This differential enzymatic regulation mechanistically explains the observed MAGN-induced increases in both GSH and GSSG pools, infer MAGN’s superior efficacy in counteracting drug-induced oxidative stress. Furthermore, intracellular 11β-HSD1 was additionally analyzed. This enzyme serves as a critical regulator in the metabolic of 2,5-hydroxyvitamin D2 and prednisolone. Functioning predominantly as a reductase, 11β-HSD1 catalyzes the regeneration of active glucocorticoids via prednisone-to-prednisolone conversion, thereby amplifying glucocorticoid receptor signaling and indirectly elevating intracellular 2,5-hydroxyvitamin D2 levels [60]. As demonstrated in Figure 7C, DIAM induction elicited the most pronounced upregulation of 11β-HSD1 expression (*p* < 0.05). These molecular findings mechanistically account for the observed DIAM-mediated increases in both prednisolone and 2,5-hydroxyvitamin D2 concentrations, substantiating DIAM’s enhanced interventions in NAFLD and viral hepatitis.

## 4. Conclusions

The hepatoprotective effects of glycyrrhizinate derivatives, particularly MONO, DIAM, and MAGN, have been widely recognized; however, their structural similarities and overlapping therapeutic outcomes have historically obscured their distinct clinical utilities. This study pioneers the application of cellular metabolomics to unravel the differential mechanisms of DIAM and MAGN, uncovering novel insights into their unique pathways of action. A key unreported finding is the superior capacity of MAGN to augment intracellular antioxidant reserves, notably elevating reduced GSH and oxidized GSSG levels compared to DIAM, while maintaining a stable GSH/GSSG ratio. In addition, MAGN uniquely enhanced α-Cehc, a water-soluble antioxidant metabolite. This underscores MAGN’s unique role in counteracting drug-induced oxidative stress, a mechanism not previously attributed to its stereoisomeric structure. Conversely, DIAM demonstrated pronounced efficacy in modulating immune-related metabolites. DIAM significantly upregulated metabolites linked to viral hepatitis and metabolic dysfunction (e.g., 25-hydroxyvitamin D2 and dioscin). DIAM appears to be more suited for addressing NAFLD and viral hepatitis. By exploring the intricate mechanisms governing these pathways and their associated metabolites, we aspire to contribute novel strategies and methodologies that can inform the prevention and treatment of liver diseases. It is also noteworthy that the current findings were derived from in vitro experiments utilizing LO2 cells, which may not fully recapitulate the intricate pathophysiological complexity of the human liver. Additionally, APAP, as a single hepatotoxic agent, may not comprehensively represent the diverse mechanisms of other clinically relevant hepatotoxins. Future investigations should prioritize evaluating the long-term effects of various commonly misused drugs in liver injury. Furthermore, integrating multi-omics methodologies—such as proteomics and transcriptomics—could establish a systematic framework to differentiate structurally analogous therapeutics, enabling a deeper exploration of upstream regulatory drivers underlying these metabolic perturbations and advancing precision medicine strategies in hepatology.

## Figures and Tables

**Table 1:** Pharmacokinetic parameters of MONO, DIAM, and MAGN (*n* = 3).

	MONO	DIAM	MAGN
AUC_0-t_ (μg·h·mL^−1^)	320.23 ± 78.63	358.04 ± 56.36	552.31 ± 119.93 *
*C*_max_ (μg·mL^−1^)	14.99 ± 3.64	18.86 ± 2.03	23.19 ± 4.18 *
*T*_max_ (h)	8 ± 0	6 ± 0	9.33 ± 2.31 ^#^
*t*_1/2_ (h)	10.82 ± 0.66	11.57 ± 1.29	23.71 ± 2.44 ***^,###^

* *p* < 0.05, *** *p* < 0.001 vs. MONO; ^#^ *p* < 0.05, ^###^ *p* < 0.001 vs. DIAM. One-way ANOVA with Tukey’s post hoc test.

**Table 2:** Parameters of OPLS-DA model.

	R2X	R2Y	Q2
Positive	0.984	0.956	0.876
Negative	0.945	0.907	0.874

**Table 3:** List of differential metabolites of DIAM and MAGN. (*n* = 6).

NO.	Description	Formular	RT (min)	Ion Mode	Highest	*p* Value	VIP	FC
1	Floctafenine	C_20_H_17_F_3_N_2_O_4_	0.72	ESI−	DIAM	0.009	1.08	4.41
2	Tacrolimus	C_44_H_71_NO_13_	0.77	ESI−	DIAM	<0.001	1.13	>10
3	Onnamide A	C_39_H_63_N_5_O_12_	0.77	ESI−	DIAM	0.037	1.03	2.42
4	Sampangine	C_15_H_8_N_2_O	0.79	ESI−	DIAM	<0.001	1.11	>10
5	Varespladib	C_21_H_20_N_2_O_5_	0.97	ESI−	DIAM	0.005	1.12	1.71
6	Ins(3,4,5,6)P4	C_6_H_16_O_18_P_4_	0.97	ESI−	DIAM	0.025	1.09	1.50
7	GSH	C_10_H_17_N_3_O_6_S	1.25	ESI+	MAGN	0.041	3.06	1.24
8	GSSG	C_20_H_32_N_6_O_12_S_2_	1.25	ESI+	MAGN	0.025	1.14	1.28
9	8-Demethyl meiqx	C_10_H_9_N_5_	1.27	ESI−	DIAM	0.023	1.02	1.92
10	Tirilazad	C_38_H_52_N_6_O_2_	3.12	ESI−	DIAM	<0.001	1.13	1.74
11	Butyroin	C_8_H_16_O_2_	3.14	ESI−	DIAM	0.026	1.04	1.23
12	Dioscin	C_45_H_72_O_16_	3.20	ESI−	DIAM	<0.001	1.03	>10
13	Prednisolone sodium tetrahydrophthalic	C_29_H_35_NaO_8_	3.70	ESI−	DIAM	<0.001	1.08	1.66
14	Ergocryptine	C_32_H_41_N_5_O_5_	3.86	ESI−	DIAM	<0.001	1.09	1.92
15	Gypsogenin	C_30_H_46_O_4_	3.90	ESI−	DIAM	0.001	1.07	1.79
16	7-Dehydrocholesterol benzoate	C_34_H_48_O_2_	4.41	ESI−	DIAM	<0.001	1.11	>10
17	Sphingosine-1-phosphate (d16:1)	C_16_H_34_NO_5_P	4.45	ESI−	DIAM	0.002	1.05	3.78
18	Argiotoxin 659	C_31_H_53_N_11_O_5_	4.46	ESI−	DIAM	0.047	1.03	2.98
19	Tisocalcitate	C_31_H_48_O_5_	4.54	ESI−	DIAM	0.001	1.08	1.39
20	Glabric acid	C_30_H_46_O_5_	4.60	ESI+	DIAM	<0.001	3.44	>10
21	Glycyrrhizin	C_42_H_62_O_16_	4.84	ESI+	DIAM	0.042	5.52	1.24
22	Propapyriogenin A2	C_30_H_44_O_5_	4.86	ESI+	DIAM	0.000	1.63	6.06
23	25-Hydroxyvitamin D2	C_28_H_44_O_2_	4.98	ESI+	DIAM	<0.001	1.79	3.85
24	Ammonium glycyrrhizate	C_42_H_65_NO_16_	4.98	ESI+	DIAM	<0.001	1.69	2.12
25	Stanozolol	C_21_H_32_N_2_O	5.36	ESI−	DIAM	0.002	1.06	1.81
26	Glycyrrhetaldehyde	C_30_H_46_O_3_	5.57	ESI+	DIAM	<0.001	1.92	>10
27	Trametenolic acid	C_30_H_48_O_3_	5.77	ESI+	DIAM	<0.001	2.52	>10
28	Cerlapirdine	C_22_H_23_N_3_O_3_S	6.33	ESI+	DIAM	0.002	1.03	>10
29	Testosterone ketolaurate	C_31_H_48_O_4_	6.33	ESI+	DIAM	<0.001	1.25	1.67
30	3-(10Z)-10-Heptadecen-1-ylphenol	C_23_H_38_O	6.86	ESI−	DIAM	0.026	1.01	1.68
31	Pristimerin	C_30_H_40_O_4_	7.38	ESI−	DIAM	0.007	1.06	1.26
32	Euscaphic acid	C_30_H_48_O_5_	10.99	ESI+	DIAM	<0.001	2.39	4.79
33	Alpha-Cehc	C_16_H_22_O_4_	11.14	ESI+	MAGN	<0.001	1.67	1.21
34	Pinnatoxin G	C_42_H_63_NO_7_	13.29	ESI+	DIAM	0.012	1.31	1.55
35	Glycerol phenylbutyrate	C_33_H_38_O_6_	15.79	ESI+	DIAM	0.013	1.27	1.36
36	Solanidine	C_27_H_43_NO	16.82	ESI+	MAGN	0.009	1.60	1.32
37	N-eicosanoylsphinganine	C_38_H_77_NO_3_	18.11	ESI+	DIAM	<0.001	1.67	2.07
38	C14 Ceramide	C_32_H_63_NO_3_	19.27	ESI+	DIAM	0.010	1.13	1.20
39	CETYL BENZOATE	C_23_H_38_O_2_	19.67	ESI+	DIAM	0.024	1.13	1.37
40	N-tetracosanoylsphinganine	C_42_H_85_NO_3_	20.30	ESI+	DIAM	0.026	1.69	>10
41	C16 Lactosylceramide (d18:1/16:0)	C_46_H_87_NO_13_	20.96	ESI+	DIAM	0.030	3.30	1.24
42	Ceramide (d18:1/16:0)	C_34_H_67_NO_3_	20.98	ESI+	DIAM	0.001	2.92	1.20
43	N-[(15Z)-tetracosenoyl]sphing-4-enine-1-phosphocholine	C_47_H_93_N_2_O_6_P	21.99	ESI+	MAGN	<0.001	1.07	2.55
44	Menaquinol-8	C_51_H_74_O_2_	22.01	ESI+	MAGN	0.009	6.20	1.68
45	Dioleoylphosphatidylserine	C_42_H_78_NO_10_P	22.01	ESI+	MAGN	0.005	1.18	1.92
46	Tetrahydrospirilloxanthin	C_42_H_64_O_2_	27.84	ESI−	DIAM	<0.001	1.08	1.29

## Data Availability

No data were used for the research described in the article.
